# BK-UM in patients with recurrent ovarian cancer or peritoneal cancer: a first-in-human phase-I study

**DOI:** 10.1186/s12885-017-3071-5

**Published:** 2017-01-31

**Authors:** Shingo Miyamoto, Fusanori Yotsumoto, Taeko Ueda, Tatsuya Fukami, Ayako Sanui, Kohei Miyata, Sung Ouk Nam, Satoshi Fukagawa, Takahiro Katsuta, Miyako Maehara, Haruhiko Kondo, Daisuke Miyahara, Kyoko Shirota, Toshiyuki Yoshizato, Masahide Kuroki, Hiroaki Nishikawa, Keijiro Saku, Yoshio Tsuboi, Kenji Ishitsuka, Yasushi Takamatsu, Kazuo Tamura, Akira Matsunaga, Toru Hachisuga, Shinsuke Nishino, Takashi Odawara, Kazuhiro Maeda, Sadao Manabe, Toyokazu Ishikawa, Yoshinobu Okuno, Minako Ohishi, Tomoya Hikita, Hiroto Mizushima, Ryo Iwamoto, Eisuke Mekada

**Affiliations:** 1 Department of Obstetrics and Gynecology, Faculty of Medicine, Fukuoka University, 7-45-1 Nanakuma, Jonan-ku, Fukuoka, 814-0180 Japan; 2 Center for Advanced Molecular Medicine, 7-45-1 Nanakuma, Jonan-ku, Fukuoka, 814-0180 Japan; 3 Department of Obstetrics and Gynecology, School of Medicine, Kurume University, 67 Asahi-machi, Kurume, Fukuoka 830-0011 Japan; 4 Department of Biochemistry, Faculty of Medicine, Fukuoka University, 7-45-1 Nanakuma, Jonan-ku, Fukuoka, 814-0180 Japan; 5 Department of Cardiology, Faculty of Medicine, Fukuoka University, 7-45-1 Nanakuma, Jonan-ku, Fukuoka, 814-0180 Japan; 6 Department of Neurology, Faculty of Medicine, Fukuoka University, 7-45-1 Nanakuma, Jonan-ku, Fukuoka, 814-0180 Japan; 7 Department of Hematology and Immunology, Kagoshima University Medical and Dental Hospital, 8-35-1 Sakuragaoka, Kagoshima, 890-8544 Japan; 8 Department of Internal Medicine, Division of Medical Oncology, Hematology and Infectious Disease, Faculty of Medicine, Fukuoka University, 7-45-1 Nanakuma, Jonan-ku, Fukuoka, 814-0180 Japan; 9 Department of Laboratory Medicine, Faculty of Medicine, Fukuoka University, 7-45-1 Nanakuma, Jonan-ku, Fukuoka, 814-0180 Japan; 10 Department of Obstetrics and Gynecology, University of Occupational and Environmental Health School of Medicine, 1-1 Iseigaoka, Yahatanishi-ku, Kitakyushu, 807-8555 Japan; 11 Kanonji Institute, Research Foundation for Microbial Diseases of Osaka University, 2-9-41 Yahata-Cho, Kanonji, Kagawa 768-0061 Japan; 12 Department of Cell Biology, Research Institute for Microbial Disease, Osaka University, 3-1 Yamadaoka, Suita, Osaka 565-0871 Japan; 13 Department of Biochemistry, School of Pharmaceutical Sciences, University of Shizuoka, 52-1 Yada, Suruga-ku, Shizuoka, 422-8526 Japan

**Keywords:** BK-UM, HB-EGF, Ovarian cancer, Phase-I study, Targeted therapy

## Abstract

**Background:**

BK-UM (CRM197) is a mutant form of diphtheria toxin and a specific inhibitor of heparin-binding epidermal growth factor-like growth factor (HB-EGF). We assessed the safety, pharmacokinetics, recommended dose, and efficacy of BK-UM in patients with recurrent ovarian cancer (OC) or peritoneal cancer (PC), and measured HB-EGF levels in serum and abdominal fluid after BK-UM administration.

**Methods:**

Eleven patients with advanced or recurrent OC or PC were enrolled and treated with BK-UM *via* the intraperitoneal route. The dose was escalated (1.0, 2.0, 3.3, and 5.0 mg/m^2^) using a 3 + 3 design.

**Results:**

Eight of 11 patients completed treatment. No dose-limiting toxicity (DLT) was experienced at dose levels 1 (1.0 mg/m^2^) and 2 (2.0 mg/m^2^). Grade 3 transient hypotension as an adverse event (defined as a DLT in the present study) was observed in two of four patients at dose level 3 (3.3 mg/m^2^). Treatment with BK-UM was associated with decreases in HB-EGF levels in serum and abdominal fluid in seven of 11 patients and five of eight patients, respectively. Clinical outcomes included a partial response in one patient, stable disease in five patients, and progressive disease in five patients.

**Conclusions:**

BK-UM was well tolerated at doses of 1.0 and 2.0 mg/m^2^, with evidence for clinical efficacy in patients with recurrent OC or PC. A dose of 2.0 mg/m^2^ BK-UM is recommended for subsequent clinical trials.

**Trial registration:**

This trial was prospectively performed as an investigator-initiated clinical trial. The trial numbers are UMIN000001002 and UMIN000001001, with registration dates of 1/30/2008 and 2/4/2008, respectively. UMIN000001001 was registered as a trial for the continuous administration of BK-UM after UMIN000001002.

**Electronic supplementary material:**

The online version of this article (doi:10.1186/s12885-017-3071-5) contains supplementary material, which is available to authorized users.

## Background

Ovarian cancer (OC) is a lethal gynecologic malignancy that has an extremely poor prognosis, due to the extension of OC cells into the peritoneal cavity [1]. Metastasis from epithelial ovarian cancer (EOC) can occur *via* hematogenous, lymphatic, or transcoelomic routes, of which the last is the most common, and is responsible for the highest prevalence of morbidity and mortality in patients with OC [1]. Most patients with recurrent OC experience massive ascites and ileus associated with transcoelomic metastasis, and preventing transcoelomic metastasis may thus play a key role in improving the prognosis in OC patients.

In the past decade, cytoreductive surgery and the introduction of platinum plus taxane-based chemotherapies have improved survival in patients with EOC [2, 3]. In the International Collaborative Ovarian Neoplasm 7 trial, standard chemotherapy with bevacizumab failed to increase overall survival in patients with newly diagnosed OC, but did improve progression-free survival and retention of peritoneal fluid [4]. Platinum is recognized as a key drug for OC therapy [5], and the development of biological agents and molecular-targeted therapeutics with low toxicity are needed to overcome resistance or restore sensitivity to platinum.

Heparin-binding epidermal growth factor-like growth factor (HB-EGF) is an epidermal growth factor receptor (EGFR) ligand [6]. HB-EGF is initially synthesized as a transmembrane protein (proHB-EGF), which is then cleaved by a protease at the cell surface to yield the mitogenic-active soluble form of HB-EGF (sHB-EGF) *via* ectodomain shedding [7]. sHB-EGF is a potent mitogen that promotes cell adhesion, cell motility, and angiogenesis [8]. HB-EGF also contributes to resistance to chemotherapy [9]. HB-EGF expression was shown to be enhanced in cancer tissues or peritoneal tissues in OC patients [10–13], and high expression of HB-EGF in cancer tissues was significantly associated with a poor prognosis in OC [10]. Emerging evidence suggests that HB-EGF is a rational therapeutic target for cancers, including OC, gastric cancer, and breast cancer [14, 15].

CRM197 is the active ingredient of the investigational drug BK-UM. CRM197 was originally isolated as a non-toxic mutant form of diphtheria toxin (DT), and has since been shown to inhibit the proliferation of cancer cells by three modes of action [16–18]. CRM197 strongly inhibited tumor growth in nude mice in various cell systems [10]. However, CRM197 displays 10^6^-fold less toxicity than DT [17], which is recognized as being extremely toxic, Massive release of DT is likely to cause lethal necrosis in the heart and liver, with a reported lethal dose in humans of about 0.1 μg/kg body weight [19]. It is therefore necessary to monitor any potential severe adverse effects of BK-UM, including cardio-, neuro-, or nephrotoxicities, irrespective of its route of administration.

CRM197 was safe and relatively well tolerated when injected subcutaneously *via* the abdominal wall on alternate days for 6 days at 1.7, 2.6, and 3.5 mg/day in patients with recurrent cancer [20]. In a pre-clinical study, we reported that daily intraperitoneal administration of BK-UM, which has demonstrated a potentially dose-dependent anti-cancer effect, may be the optimal regimen [21]. Herein, we report the results of a phase-I study designed to assess the safety, pharmacokinetics, recommended phase-II dose, and efficacy of BK-UM in patients with recurrent OC or peritoneal cancer (PC).

## Methods

### Patient selection

This clinical trial was undertaken as an open-label, phase-I dose-escalation study, and was performed as a prospective investigator-initiated clinical trial. The study was conducted at Fukuoka University Hospital (Fukuoka, Japan) in accordance with the principles of the Declaration of Helsinki and the International Conference on Harmonization’s Good Clinical Practice Guidelines. The study protocol was approved by the Institutional Review Board of Fukuoka University Hospital.

Study-inclusion criteria were:(i) EOC or primary PC confirmed by histology; (ii) recurrent OC or PC within 6 months after completion of a primary treatment containing platinum and a taxane (including patients who had received second- or subsequent-line chemotherapy after recurrence following primary treatment containing platinum and a taxane); (iii) performance status 0 or 1 according to the Eastern Cooperative Oncology Group classification; (iv) evidence of adequate liver, kidney, and bone marrow functions (absolute white blood cell count >3000/μL, or neutrophil count ≥1000/μL, platelet count ≥100,000/μL, hemoglobin ≥8.0 g/dL, aspartate aminotransferase ≤2.5-fold the upper limit of normal, alanine transaminase ≤2.5-fold the upper limit of normal, serum total bilirubin ≤1.5 mg/dL, serum creatinine ≤1.5 mg/mL); (v) detectable levels of HB-EGF in serum or peritoneal fluid; (vi) diphtheria antibody titer <0.1 IU/mL; and (vii) practicality of placing a port in the abdomen.

All subjects underwent electrocardiography to confirm the absence of abnormalities requiring treatment, and sensory and motor nerve-conduction studies to evaluate peripheral neuropathy.

Exclusion criteria were: (i) history of cardiac disease ≥ III according to the New York State Heart Association classification; (ii) <50% left ventricular ejection fraction by echocardiography; (iii) myocardial infarction within 6 months of study enrollment; (iv) uncompensated liver cirrhosis; (v) severe lung disease necessitating oxygen inhalation; (vi) psychotic disorders; (vii) uncontrolled diabetes mellitus; (viii) ileus or subileus; (ix) uncontrolled infectious disease; (x) peripheral neuropathy of grade ≥2; (xi) pregnancy or breastfeeding; (xii) life expectancy <3 months; (xiii) receipt of an investigational agent within 28 days before study enrollment; and (xiv) history of administration of antiserum.

Concurrent investigational or antineoplastic agents were not permitted during the study.

### Investigational drug

CRM197 was produced from *Corynebacterium diphtheria* C7β (197) and purified by the Kanonji Institute at the Research Foundation for Microbial Diseases of Osaka University (Osaka, Japan). BK-UM contained CRM197, sodium phosphate, and sucrose. BK-UM was also produced at the above institute according to good manufacturing practice.

### Study design

This was a dose-escalation study with a 3 + 3 trial design at each dose to determine the maximum-tolerated dose (MTD) and recommended dose. BK-UM was administered intraperitoneally at doses of 1.0, 2.0, 3.3, and 5.0 mg/m^2^, 5 days a week for 2 weeks, based on the results of a pre-clinical study [21]. An anti-histamine was administered as premedication before BK-UM injection. Dose escalation of BK-UM was investigated by the Data and Safety Monitoring Committee (DSMC). Treatment of the first patient was finished completely, and the safety of BK-UM was then reviewed by the DSMC. If no dose-limiting toxicity (DLT) was observed in three patients at a given dose, the next three patients were treated with the next-higher dose. If DLT was experienced by one or two of the three patients at any dose, three additional patients were treated with the same dose. If two or fewer of six patients treated with a given dose exhibited DLT, this dose was considered to exceed the MTD and dose escalation was halted. The recommended phase-II dose was defined as one dose level lower than the MTD. Patients who progressed after showing a response to BK-UM were not allowed to be re-treated with BK-UM. If more than two patients withdrew from the present study, safety was evaluated by the DSMC. Conventional chemotherapy was administered in patients who requested it after the observation period of the trial, for as long as there was a diagnosis of progressive disease, or for six courses in patients with stable disease (SD), partial response (PR), or complete response.

### Safety evaluation and definition of DLT

Safety was evaluated every week during treatment and until 6 weeks after the first infusion of BK-UM. Safety of BK-UM was evaluated according to adverse events (AEs) detected *via* vital signs, physical signs, whole blood cell counts, serum chemistry, urinalyses, 12-lead electrocardiography, cardiac monitoring, echocardiography, muscle tests, neural reflex tests, nerve conduction velocity testing, and thoracoabdominal radiographs. Laboratory tests for in cardiotoxicity were also undertaken.

All AEs were graded according to the Common Terminology Criteria for Adverse Events v3.0 set by the National Cancer Institute. DLT was defined as an AE or laboratory abnormality that occurred 6 weeks after the first infusion of BK-UM. A DLT was judged to be related to BK-UM if it met any of the following criteria: hematologic toxicity grade ≥4; gastrointestinal disorder possibly due to peritonitis carcinomatosa grade ≥4; or non-hematologic toxicity grade ≥3. Safety data were evaluated by the DSMC at all doses.

### Efficacy assessments

Responses were evaluated 6 weeks after the first infusion of BK-UM. Recurrent lesions were evaluated by computed tomography before infusion of BK-UM, to provide baseline images. Clinical evaluation of response was estimated using baseline computed tomography images and findings 6 weeks after the first infusion of BK-UM, according to Response Evaluation Criteria in Solid Tumors (RECIST) v1.0 guidelines. The interval for overall survival was defined as the period from the day of the first infusion of BK-UM to the day on which death occurred. Some patients were discharged from Fukuoka University Hospital after the end of this clinical trial and treated at other hospitals, and the dates of death for these patients were recorded using information acquired from those hospitals.

### Pharmacokinetics

Blood samples were collected before and at 1, 3, 6, and 12 h after infusion of each dose on day 1. Pre-dose samples were collected on days 2–5 and 8–12. Samples were collected in a heparinized tube and centrifuged at 3000 × *g* for 10 min at room temperature. Plasma samples were stored at −80 °C for analysis of BK-UM concentrations. Plasma concentrations of BK-UM were determined by enzyme-linked immunosorbent assay (ELISA). Plates were coated with an anti-DT monoclonal antibody to capture CRM197, followed by the addition of diluted plasma samples obtained from patients, and a horseradish peroxidase-labeled monoclonal antibody against DT. The sensitivity of this assay was ≥20 ng/mL.

### Measurement of HB-EGF levels in serum and abdominal fluid

Blood and abdominal fluid were withdrawn from all patients into anticoagulant-excluded tubes before and after infusion of BK-UM. HB-EGF concentrations in serum and abdominal fluid were determined by a modified enzyme assay [22] using a sandwich ELISA (DuoSet; R&D Systems, Minneapolis, MN, USA).

## Results

### Patient characteristics

This trial was conducted at 12/10/2007. Eleven of 50 patients with recurrent cancer were enrolled, including 10 with OC and one with primary PC (Table 1). The remaining 39 patients were not eligible for the following reasons: 12 patients had diphtheria antibody levels >0.1 IU/mL; 21 and five patients had performance statuses of 2 or 3, respectively; and one patient recurred more than 6 months after primary treatment containing platinum and a taxane. Among the patients who received BK-UM at 1.0 mg/m^2^ (level 1 dose), one patient had leptomeningeal disease previously treated using a γ-knife, but had been in a stable condition for 4 months. Four patients (numbers 1–4) were enrolled at 1.0 mg/m^2^ BK-UM (dose level 1) because one patient who lived in Tokyo wanted to return to Tokyo, and withdrew their consent soon after BK-UM treatment.

**Table 1:** Clinical characteristics

Patient no.	Cancer subtype	Previous chemotherapy	Previous radiotherapy	PS
1	OVCA (EMC)	5 lines	None	1
2	OVCA (SCC)	2 lines	None	1
3	OVCA (SCC)	2 lines	None	1
4	OVCA (SCC)	1 lines	γ-knife	1
5	PC (SCC)	2 lines	None	1
6	OVCA (SCC)	3 lines	None	1
7	OVCA (SCC)	8 lines	None	0
8	OVCA (SCC)	7 lines	None	1
9	OVCA (SCC)	4 lines	None	1
10	OVCA (CCC)	5 lines	None	1
11	OVCA (TCC)	4 lines	None	0

Age-range of the patients is 46 to 70 years

*OVCA* ovarian cancer, *EMC* endometrioid adenocarcinoma, *SCC* serous cystadenocarcinoma, *PC* peritoneal cancer, *CCC* clear cell carcinoma, *TCC* transitional cell carcinoma, *PS* performance status

Eight patients completed the study according to the protocol, one patient withdrew their consent as noted above, and two patients dropped out of the trial because of DLTs. All 11 enrolled patients were evaluated for safety and efficacy on an intent-to-treat basis.

### Safety and tolerability

All AEs of grade ≥2 are listed in Table 2. All patients experienced at least one AE. At BK-UM doses of 1.0 and 2.0 mg/m^2^ (dose levels 1 and 2), all AEs were considered unlikely to have a causal association with BK-UM. Grade 3 AEs such as ileus, abdominal fullness, and anemia were observed in one patient at 2.0 mg/m^2^ BK-UM (dose level 2). Most patients had grade 1 fever between days 1 and 3 of BK-UM treatment, but neither the frequency nor severity of these AEs increased with dose escalation. BK-UM was thus generally considered to be well-tolerated at doses of 1.0 and 2.0 mg/m^2^. However, both patients treated at 3.0 mg/m^2^ BK-UM (dose level 3) developed grade 3 hypotension on day 8 of treatment, which was defined as a DLT. This AE manifested as nausea, abdominal pain, and transient hypotension, accompanied by irritation of the peritoneum, fever, and elevated C-reactive protein levels. Complete recovery from all AEs was observed within 1 week in both patients. All other AEs observed at dose level 3 were grade ≤2. No severe AEs (grades 4 or 5), including severe cardiotoxicity- or neurotoxicity-related DLTs, were found in any patient at any dose level.

**Table 2:** Adverse events by grade in this phase-I study

	Level 1				Level 2			Level 3			
Patient no.	1	2	3	4	5	6	7	8	9	10	11
Appetite loss	2	2									
General fatigue									2		
Nausea		2							2		
Vomiting		2							2		
Ileus		2				3					
Constipation	2					2			2		
Diarrhea						2					
Abdominal pain		2				2					
Abdominal fullness	2					3					
Intraabdominal bleeding										2	
Lumbago						2					
Headache						2					
Fever						2^c^	2^c^				
Dyspnea									2		
Hematoma^a^							2				
Erythema									2		
Anemia	2			2	2	3	2				
Elevation of ALT							2				
Elevation of CRP			2				2^c^	2^c^			
Hypoalbuminemia	2		2								
Hypotension^b^								3^c^			3^c^

^a^Hematoma in the inserted port

^b^Transient hypotension accompanied by abdominal peritoneal irritation

^c^Adverse events during the administration of BK-UM

No asterisk in adverse events indicates events monitored during the observation period after the administration of BK-UM

### Dose escalation and MTD

No DLT was observed at dose levels 1 and 2, but two patients at dose level 3 showed grade 3 hypotension 8 days after the first infusion. The MTD was therefore judged to be at dose level 3 (3.0 mg/m^2^), and the recommended dose was determined to be 2.0 mg/m^2^.

### Pharmacokinetics

The plasma concentration of BK-UM after intraperitoneal administration was measured 15 times per course in each patient. At 1.0 mg/m^2^ BK-UM (dose level 1), plasma BK-UM was only detected in three of 60 samples from four patients. BK-UM plasma levels were detected in nine of 45 samples from three patients at 2.0 mg/m^2^ (level 2) and 11 of 51 samples from four patients at 3.0 mg/m^2^ (level 3). The plasma concentration of BK-UM was unmeasurable throughout the treatment with BK-UM in five patients, while BK-UM was only sporadically detected in plasma in the remaining six patients. These results suggested that intraperitoneal delivery of BK-UM did not result in clinically relevant plasma exposure (Additional file 1: Table S1).

### Evaluation of response

BK-UM treatment resulted in reductions in HB-EGF serum concentrations in five patients, in abdominal fluid in four patients, and in abdominal fluid and serum in four patients (Table 3). Patients 2, 8, and 11 were not included in this analysis because HB-EGF concentrations were not measured on the scheduled day after BK-UM treatment in these patients. The present results suggested that BK-UM reduced HB-EGF concentrations in abdominal fluid or serum if the HB-EGF concentration was ≥125 pg/mL. Serum levels of cancer antigen-125 also decreased in two of 11 patients. DT antibody levels were increased in all patients after BK-UM treatment. One of the 11 patients achieved PR (Table 3), five patients showed SD, and the remaining five patients showed progressive disease. In addition to the abdominal cavity, patients 4, 6, and 9 had metastatic lesions in the brain, liver, and lungs, respectively (though patient number 4 had a history of leptomeningeal disease previously treated by γ-knife, and had been in a stable condition for 4 months). The metastatic lesions in the abdominal cavity in these three patients were markedly reduced after BK-UM treatment, even though the extra-abdominal lesions had progressed or were stable. At least three patients received intraperitoneal administration of BK-UM 1 mg/m^2^ or 2 mg/m^2^ for 2 weeks. Only patients 1 and 4 (level 1) received two courses of intraperitoneal BK-UM (1 mg/m^2^), and responses in these patients were evaluated at week 8 after the start of the first course of BK-UM, compared with evaluation at week 6 in the other patients who only received one course of intraperitoneal BK-UM. The benefit of intraperitoneal administration of BK-UM thus lasted up to 6 weeks in patients 3, 5, 10, and 11, and 8 weeks in patients 1 and 4. Conventional chemotherapy was started at week 9 in patients 1 and 4, and week 7 in patients 3, 7, 8, 10, and 11. Patients 2, 5, 6, and 9 requested no further therapy. Five of the seven patients who received further conventional chemotherapy exhibited overall survival beyond 14 months (Table 4). Three patients (1, 3, and 11) showed no evidence of disease following BK-UM treatment and subsequent therapy, and one patient (11) remained free of disease.

**Table 3:** Clinical effects in this phase I study

Patient no.	HB-EGF in serum (pg/mL)		HB-EGF in abdominal fluid (pg/mL)		Serum CA125 (IU/mL)		Anti-diphtheria toxin antibodies (IU/mL)		Efficacy
	Pre-Tx	Post-Tx	Pre-Tx	Post-Tx	Pre-Tx	Post-Tx	Pre-Tx	Post-Tx	
1	1971	196	2169	106	291	137	<0.01	2.56	SD
2	556	349^a^	416	560^a^	1411	3487	<0.01	124	PD
3	297	172	593	292	46	50	<0.01	>131	SD
4	267	85	143	34	1408	2217	0.04	>131	PR
5	51	101	55	399	267	600	0.03	31.1	SD
6	74	109	109	171	254	158	<0.01	81.9	PD
7	67	79	130	ND	2813	3983	<0.01	0.06	PD
8	162	141^a^	140	132^a^	3894	5147	0.13	20.5	PD
9	231	130	237	66	1856	3984	0.01	1.02	PD
10	247	140	ND	ND	40	63	<0.01	>41.0	SD
11	67	155^a^	ND	ND	16	94	<0.01	>6.56	SD

*Pre-A* pre-administration of BK-UM, *Post-A* post-administration of BK-UM, *ND* not determined, *SD* stable disease, *PD* progressive disease, *PR* partial response

^a^HB-EGF level was measured on the final day of investigation

**Table 4:** Overall survival of patients after BK-UM administration

Patient no.	Conventional chemotherapy after BK-UM	Overall survival (months)
1	CDDP + CPT-11 (6 courses)	28.1
2	–	3.4
3	CDDP + CPT-11 (6 courses)	24.3
4	CDDP + CPT-11 (6 courses)	15.1
5	–	5.5
6	–	5.0
7	CDDP + CPT-11 (2 courses)	7.4
8	CDDP + IFM (2 courses)	4.2
9	–	3.6
10	CPT-11 (6 courses)	14.0
11	CBDCA + Doxil (6 courses)	>60^a^

*CDDP* cisplatin, *CPT-11* irinotecan, *IFM* Ifosfamide, *CBDCA* carboplatin, *Doxil* doxorubicin hydrochloride liposome injection

^a^Patient alive at 11/1/2016

## Discussion

HB-EGF is a ligand that binds to the EGFR. Although several studies have investigated anti-cancer agents targeting the EGFR and other ERBB family receptors, few clinical trials have investigated the use of agents targeting EGF family growth factor ligands [23]. Previous studies have suggested that HB-EGF is expressed at high levels in OC patients, and represents a promising target for OC treatment [10, 14]. However, the current study provides the first evidence of HB-EGF values in OC patients.

A previous phase-I study of intraoperative intraperitoneal administration of 60–100 mg/m^2^ cisplatin detected a maximum plasma concentration of cisplatin of 0.7–2.3 μg/mL after 1–1.5 h, giving a mean peritoneal-to-plasma area-under-the-curve ratio during perfusion of 19.5 for 100 mg/m^2^ [24]. The plasma cisplatin level ranged from one-third to one-tenth of the total amount of intraperitoneally administered cisplatin. Conversely, the plasma BK-UM level was >20 ng/mL. Intraperitoneal administration of 1–3.3 mg/m^2^ BK-UM would be expected to result in plasma levels of ≥100 ng/mL; however, BK-UM was undetectable in most plasma samples in the current study. There are several possible reasons for this phenomenon: (i) BK-UM may not pass through peritoneal membranes readily because of its high molecular weight; (ii) BK-UM may be absorbed readily into the lymphatic system in the peritoneum; and (iii) most BK-UM may bind rapidly to the peritoneum, with high expression of HB-EGF on peritoneal membranes. In the present study, even patients with DLTs had undetectable plasma levels of BK-UM, suggesting that AEs are induced by local administration of BK-UM.

Hypersensitivity was mitigated by administration of an anti-histamine in all patients before intraperitoneal administration of BK-UM. Although fever (a common AE) occurred up to 3 days after the beginning of BK-UM administration, it was not accompanied by general fatigue, suggesting that the fever was generated locally. Other AEs, including nausea, vomiting, appetite loss, and abdominal fullness, could have been related to recurrent disease. Two patients treated at dose level 3 developed transient hypotension as a DLT at day 8, associated with uncontrolled abdominal pain and elevated C-reactive protein levels. Two patients with DLTs at dose level 3 did not have ascites, whereas the other two patients who did have ascites had no severe AEs. Transient hypotension could have been mediated by a parasympathetic reaction related to stimulation of the peritoneum by BK-UM, which could have caused inflammation in the abdominal cavity. It was also notable that both patients developed DLT hypotension at day 8. BK-UM suppresses the function of HB-EGF in the peritoneum, suggesting that it may be associated with blocking the circulation of peritoneal fluid, and high concentrations of BK-UM may thus cause excessive peritoneal stimulation. In a phase-I study of a monoclonal antibody (KHK2866) against HB-EGF, neurotoxicity manifested as complex partial seizures, aphasia, and confusion after first-dose intravenous administration, but these AEs were reversible and unpredictable [23, 25]. In the present study, muscle tests, neural reflex tests, and nerve conduction velocity tests revealed no neurotoxic abnormalities in any patient. However, in the event of intravenous administration of BK-UM, patients should be closely monitored for severe AEs such as cardiotoxicity, neurotoxicity, and nephrotoxicity.

The present study enrolled patients who had progressed to advanced disease despite previous treatments with several chemotherapeutic regimens. In addition, safety evaluations were performed 8 weeks after enrollment in patients who received one cycle of the present study treatment. Chemotherapeutic regimens are usually repeated for 3–4 weeks in OC patients, and demonstrating clinically favorable efficacy of BK-UM would thus be difficult because of the long observation period in the current study. However, one of five patients achieved PR or SD, suggesting that BK-UM may be effective against peritoneal dissemination of OC. Seven of 11 patients were treated with conventional chemotherapy after intraperitoneal administration of BK-UM, five of whom survived for >14 months. These five patients also had PR or SD, and four of them received another platinum-based chemotherapeutic regimen. In principle, the prognosis of OC depends on the tumor’s sensitivity to platinum [5], and patients who are refractory or resistant to platinum treatment showed a sensitivity of <10% to second-line chemotherapy [5]. These results suggest that BK-UM may represent a promising anti-cancer agent for OC therapy, by rescuing platinum sensitivity by improving peritoneal dissemination. Conversely, intrahepatic lesions or pleural effusions were exacerbated by intraperitoneal administration of BK-UM, though it is possible that replacing local intraperitoneal therapy with systemic intravenous administration of BK-UM could improve intrahepatic lesions or pleural effusions.

HB-EGF levels in serum have been shown to be correlated with levels in abdominal fluid and cancer tissues in OC patients [22]. However, HB-EGF is cleaved by a disintegrin and metalloproteinase (ADAM) and other membrane proteases, including membrane-type 1 matrix metalloprotease (MT1-MMP) [26]. However, the form of HB-EGF measured by ELISA in the current study was only cleaved by ADAM (preliminary results), while the form of HB-EGF cleaved by ADAM and MT-MMPs can be blocked by BK-UM [26]. In our study, BK-UM treatment elicited SD in some patients with low HB-EGF levels. Levels of the active form of HB-EGF might be elevated in patients with recurrent OC. The development of a kit for measuring all forms of HB-EGF is needed to facilitate further studies of BK-UM.

## Conclusion

The results of this first-in-human study demonstrated that BK-UM is safe and well tolerated for the treatment of OC, with an MTD and recommended dose for phase-II studies of 3.3 mg/m^2^ and 2.0 mg/m^2^, respectively. Further investigations should focus on the: (i) route of administration of BK-UM; (ii) the development of a kit for measuring associated targeted molecules; and (iii) the combination of conventional anti-cancer agents with BK-UM.

## Additional file

Additional file 1:
**Table S1.** Pharmacokinetics of BK-UM. (PDF 48 kb)

## Abbreviations

ADAM: A disintegrin and metalloproteinase
AE: Adverse event
DLT: Dose-limiting toxicity
DT: Diphtheria toxin
EGFR: Epidermal growth factor receptor
ELISA: Enzyme-linked immunosorbent assay
EOC: Epithelial ovarian cancer
HB-EGF: Heparin-binding epidermal growth factor-like growth factor
MTD: Maximum-tolerated dose
MT-MMP: Membrane-type matrix metalloprotease
OC: Ovarian cancer
PC: Peritoneal cancer
PR: Partial response
SD: Stable disease
sHB-EGF: Soluble form of heparin-binding epidermal growth factor-like growth factor
