# Supplementary material for: BK-UM in patients with recurrent ovarian cancer or peritoneal cancer: a first-in-human phase-I study
Source: BMC Cancer. 2017 Jan 31;17:89. doi: 10.1186/s12885-017-3071-5 (PMC5286856; doi:10.1186/s12885-017-3071-5)
Supplement: Additional file 1: — Table S1. Pharmacokinetics of BK-UM. (PDF 48 kb) [file 12885_2017_3071_MOESM1_ESM.pdf]

Additional Table 1. Pharmacokinetics of BK-UM

		Level 1				Level 2			Level 3			
Patient no.		1	2	3	4	5	6	7	8	9	10	11
Day 1	Pre-A	-	-	-	-	-	-	-	-	-	-	-
	Post-A	-	-	-	-	-	-	-	-	-	-	-
	1 h	-	-	-	-	-	-	-	-	-	-	-
	3 h	-	-	-	-	-	-	-	39.6	24.6	-	-
	6 h	-	-	-	-	-	-	-	57.6	29.3	-	-
	12 h	-	-	-	-	-	-	-	42.2	21.8	-	-
Day 2	Pre-A	-	-	-	-	-	-	-	-	21.7	-	-
Day 3	Pre-A	-	-	-	22.1	132	-	-	-	-	-	-
Day 4	Pre-A	-	-	-	-	162	-	-	-	21.8	-	-
Day 5	Pre-A	-	-	-	33.2	160	-	-	-	-	-	-
Day 6	Pre-A	-	-	-	-	160	-	-	-	-	-	-
Day 9	Pre-A	-	-	-	-	-	39.3	-	-	-	-	
Day 10	Pre-A	-	-	20.0	-	-	77.4	-		22.5	-	
Day 11	Pre-A	-	-	-	-	-	51.8	-		22.2	-	
Day 12	Pre-A	-	-	-	-	-	51.1	-		21.4	-	
Day 13	*	-	-	-	-	-	21.6	-		-	-	

Pre-A pre-administration of BK-UM; *Post-A* post-administration of BK-UM

Values given as ng/mL

*Sampled at the same time as day 2–12

- indicates below the measurable value
